# Mitochondrial Fusion Is Essential for Steroid Biosynthesis

**DOI:** 10.1371/journal.pone.0045829

**Published:** 2012-09-21

**Authors:** Alejandra Duarte, Cecilia Poderoso, Mariana Cooke, Gastón Soria, Fabiana Cornejo Maciel, Vanesa Gottifredi, Ernesto J. Podestá

**Affiliations:** 1 Instituto de Investigaciones Biomédicas (INBIOMED), Department of Human Biochemistry, School of Medicine, University of Buenos Aires-CONICET, Buenos Aires, Argentina; 2 Fundación Instituto Leloir-CONICET, University of Buenos Aires, Buenos Aires, Argentina; Institut de Génomique Fonctionnelle de Lyon, France

## Abstract

Although the contribution of mitochondrial dynamics (a balance in fusion/fission events and changes in mitochondria subcellular distribution) to key biological process has been reported, the contribution of changes in mitochondrial fusion to achieve efficient steroid production has never been explored. The mitochondria are central during steroid synthesis and different enzymes are localized between the mitochondria and the endoplasmic reticulum to produce the final steroid hormone, thus suggesting that mitochondrial fusion might be relevant for this process. In the present study, we showed that the hormonal stimulation triggers mitochondrial fusion into tubular-shaped structures and we demonstrated that mitochondrial fusion does not only correlate-with but also is an essential step of steroid production, being both events depend on PKA activity. We also demonstrated that the hormone-stimulated relocalization of ERK1/2 in the mitochondrion, a critical step during steroidogenesis, depends on mitochondrial fusion. Additionally, we showed that the SHP2 phosphatase, which is required for full steroidogenesis, simultaneously modulates mitochondrial fusion and ERK1/2 localization in the mitochondrion. Strikingly, we found that mitofusin 2 (Mfn2) expression, a central protein for mitochondrial fusion, is upregulated immediately after hormone stimulation. Moreover, Mfn2 knockdown is sufficient to impair steroid biosynthesis. Together, our findings unveil an essential role for mitochondrial fusion during steroidogenesis. These discoveries highlight the importance of organelles’ reorganization in specialized cells, prompting the exploration of the impact that organelle dynamics has on biological processes that include, but are not limited to, steroid synthesis.

## Introduction

Steroid hormones are synthesized in steroidogenic cells of the adrenal gland, ovary, testis, placenta, and brain and are required for normal reproductive function and body homeostasis. Steroid synthesis is regulated by trophic hormones, specifically, adrenocorticotropin hormone (ACTH) in adrenocortical cells and luteinizing hormone (LH) in testicular Leydig and ovarian cells respectively. These hormones activate G protein-coupled receptors resulting in the activation of adenylyl cyclase and an increase in intracellular cAMP levels [1]. This increase promotes the activation of cAMP-dependent protein kinase (PKA), protein synthesis and protein phosphorylation. All these processes contribute to the delivery of cholesterol from the outer to the inner mitochondrial membrane, the rate-limiting step in steroid production [2], [3].

The mitochondria are central during steroidogenesis since the physical protein-protein interactions between key factors during the transport of cholesterol takes place in the contact sites between the two mitochondrial membranes [4]. Several proteins, such as PKA, MEK and extracellular signal-regulated kinases (ERK1/2) [5], [6], which are essential to complete steroidogenesis, form a mitochondria-associated complex. However, no mitochondrial targeting sequence has been described for these protein kinases. Steroid production involves protein pospho/dephosphorylation as a balance of protein quinases and phosphatases activity. We extensively studied the role of protein tyrosine phosphatases (PTPs) in the regulation of steroid biosynthesis. We have previously demonstrated that PTP inhibitors, reduced hormone-, or cAMP-induced stimulation of steroid production [7], [8], [9]. Recently published results indicated that PTP src homology 2-containing phosphotyrosine phosphatase 2 (SHP2) is essential for steroidogenesis [10]. In addition, our group has described another essential protein in hormone-dependent steroid biosynthesis, an Acyl-CoA Synthetase 4 (Acsl4). Both Acsl4 and SHP2 are implicated in cholesterol transport into the mitochondria [7], [8].

Steroid synthesis is initiated at the inner mitochondrial membrane (IMM), where the cytochrome P450 cholesterol side chain cleavage enzyme (CYP11A1) catalyzes the conversion of cholesterol to pregnenolone [11]. Then pregnenolone enters the endoplasmic reticulum (ER) where further enzymatic reactions occur. Afterwards, the steroid formed returns to the mitochondrion to produce the final steroid hormone. Remarkably, it is widely accepted that the translocation of cholesterol from the outer mitochondrial membrane (OMM) to the IMM is the rate-limiting step in the production of all steroids [12], [13]. Therefore, the ability of cholesterol to move into mitochondria to be available for CYP11A1 determines the efficiency of steroid production.

Mitochondrial fusion/fission events, a mechanism also referred as “mitochondrial dynamics” [14], are important for maintaining the integrity of these organelles. Mitochondrial dynamics allows mitochondrial replication, repair of defective mitochondria, selective elimination of depolarized mitochondria via mitophagy and propagation of intra-mitochondrial calcium waves [15]. It has been described that mitochondrial plasticity facilitates the movement of these organelles within the cell [16], [17], and that mitochondrial rearrangements are important for the normal function of the cell, and protection against ageing-related changes [18]. In addition, it has been described that mitochondrial fission is related to metabolic disorders such as hyperglycemia [19], [20]. The above-mentioned findings demonstrate that mitochondrial dynamics plays an important role in many cellular functions. Despite the importance of this process, the mechanistic details of the regulation of mitochondrial fission-fusion dynamics remains to be completely elucidated, particularly is not well characterized in hormonal regulation of cellular functions. It has been proposed that two dynamin-like GTPases involved in mitochondrial fusion, Mitofusin (Mfn) 1 and 2 are implicated in the modulation of mitochondria-mitochondria and ER-mitochondria interactions. Mfn 1 and 2 are located in the OMM mediating mitochondrial fusion in concert with another GTPase, OPA1 (optic atrophy 1), in the IMM. Mfn 1 and 2 are extensely expressed in tissues, as demonstrated in brain (mainly Mfn2), liver, adrenal glands and testis [21]. It has been proved that Mfn2 is enriched at contact sites between ER and mitochondria (mitochondria associated membrane, MAM) [22]. Even when several lines of evidence support a regulation of Mnf2 by diverse metabolic conditions as type 2 diabetes and obesity [23], modulation in Mnf2 levels has not been demonstrated under hormonal regulation of different cellular functions. Mitochondrial fission requires dynamin related protein 1 (Drp1), a cytosolic protein, that is recruited to the OMM by a poorly characterized multiprotein complex. Recent work have shown that PKA recruitment to the mitochondria resulted in mitochondrial elongation by Drp1 phosphorylation and inactivation in neurons [24].

However, despite the key role of mitochondria in steroid synthesis, there are no reports exploring the relationship between the mitochondrial dynamics and the regulation of the onset of steroidogenesis.

Herein, we studied the effect of steroidogenic hormones in the regulation of mitochondrial fusion in specialized cells. We demonstrated that steroid synthesis depends on changes in mitochondrial fusion that can be regulated in a hormone-dependent manner. Furthermore, blocking mitochondrial fusion by knocking down Mfn2 expression has a negative impact on steroid synthesis. Conversely, Mfn2 is promptly up-regulated after the steroidogenic stimuli, thus suggesting that mitochondrial dynamics might be central for steroidogenesis. The observed changes in mitochondrial fusion might also be central for the formation of the mitochondrial multiprotein complex that delivers cholesterol to the P450 system since hormone-stimulated mitochondrial rearrangement is required for the re-localization of the ERK1/2 protein to mitochondria. In addition, SHP2 modulates both mitochondrial fusion and ERK1/2 localization in mitochondria. Taken together, our findings reveal a novel role of mitochondrial fusion in the re-localization of factors that are essential for steroidogenesis. This, in turn, suggests that the fusion of organelles might represent a limiting step in the onset of processes that require transport of intermediate products between organelles.

## Results

### Progesterone Production Correlates with Mitochondrial Rearrangements

**Figure 1:**
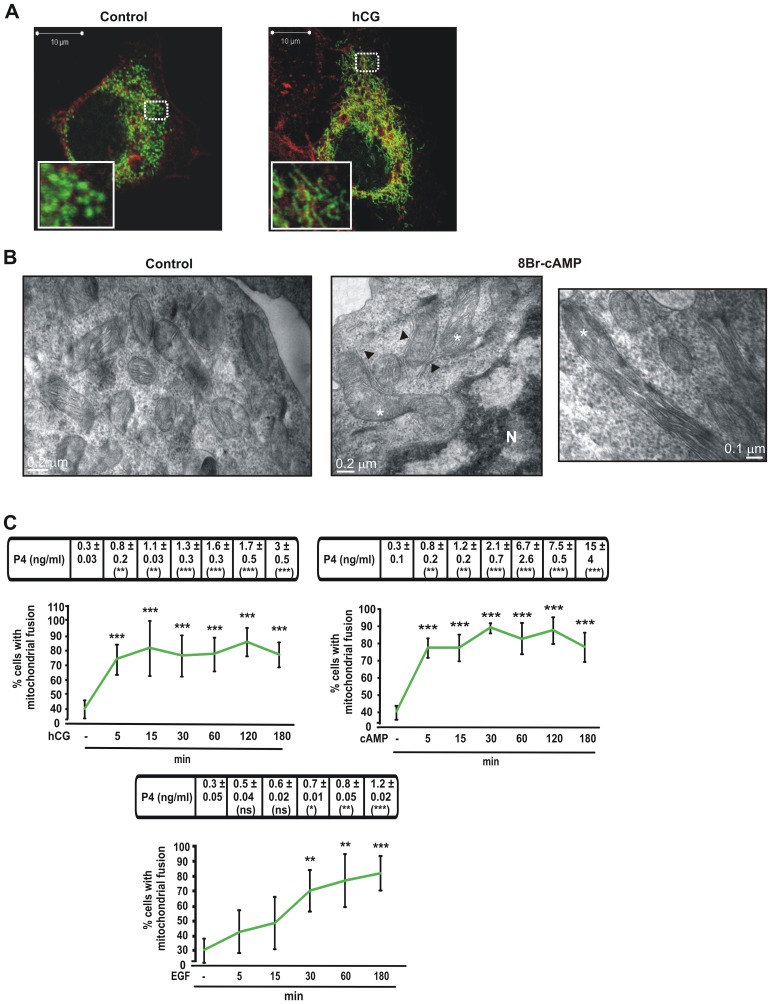
Hormonal stimulation causes an increase in fused mitochondria morphology in MA-10 cells. **A.** Representative confocal images of the MA-10 cell line transfected with mtYFP (mito, Green). After 24 h transfection, cells were stimulated with or without hCG (20 ng/ml) for 1 h. Cell morphology was visualized by actin red staining with the fluorescence dye Phalloidin–TRITC (1∶2000), incubated for 1 h at room temperature. Scale bar, 10 µm. **B.** Representative Electron micrographs of mitochondria in MA-10 cells. Cells were stimulated with or without 8Br-cAMP (1 mM) for 1 h. Scale bars, 0.2 µm. Higher magnification of another section is shown for 8Br-cAMP-treated cells (left panel). Scale bar; 0.1 µm. Asterisks indicate tubular, elongated mitochondria. Arrowheads indicate ER. **C.** After 24 h transfection with mt-YFP, MA-10 cells were treated with or without hCG (20 ng/ml), 8Br-cAMP (1 mM) or EGF (10 ng/ml), for the indicated times. Cells were processed as described in materials and methods and observed with a fluorescence microscope. Cells with the indicated mitochondrial morphology shown in right image of panel A, named as mitochondrial fusion in the graphics, were quantified. More than a hundred cells were counted manually in at least four distinct optical fields. Quantitative analysis of fused mitochondria is shown. Results are expressed as the means ± SEM of three independent experiments. ***P<0.001 vs. control. **P<0.01 vs. control. Cellular medium was used to determine P4 production by RIA. Results of P4 measurement are indicated at the top of each graph as the means ± SEM of three independent experiments. ns *P*>0.05 vs. control. ***P<0.001 vs. control. **P<0.01 vs. control.

To investigate changes in mitochondrial morphology after stimulation of steroid synthesis, we transiently transfected MA-10 Leydig cells with mitochondria-targeted YFP (mt-YFP) [25] and then stimulated them with human chorionic gonadotropin (hCG) or with 8Br-cAMP (cAMP), a cell permeable analogue of the second messenger. We evaluated mitochondrial rearrangement based on a described characterization of multiple mitochondrial shapes [26]. Under experimental conditions, we clearly distinguished two of these categories: punctuated and fused. We observed that control cells presented mainly a punctuated pattern that changed to the fused type after hormonal stimulation (Figure 1A). Electron microscopy confirmed the morphology changes we observed by fluorescence probing. In basal conditions mitochondria have an orthodox structure with narrow cristae and a more spherical shape in agreement with the punctuated shape category we described above. In hormone-stimulated cells mitochondria are larger in diameter and appeared elongated and tubular as compared to the round mitochondria found in control cells. Interestingly, ultrastructure showed that large portions of filamentous ER appeared in close proximity of enlarged mitochondria in steroid producing cells (Figure 1B).

**Figure 2:**
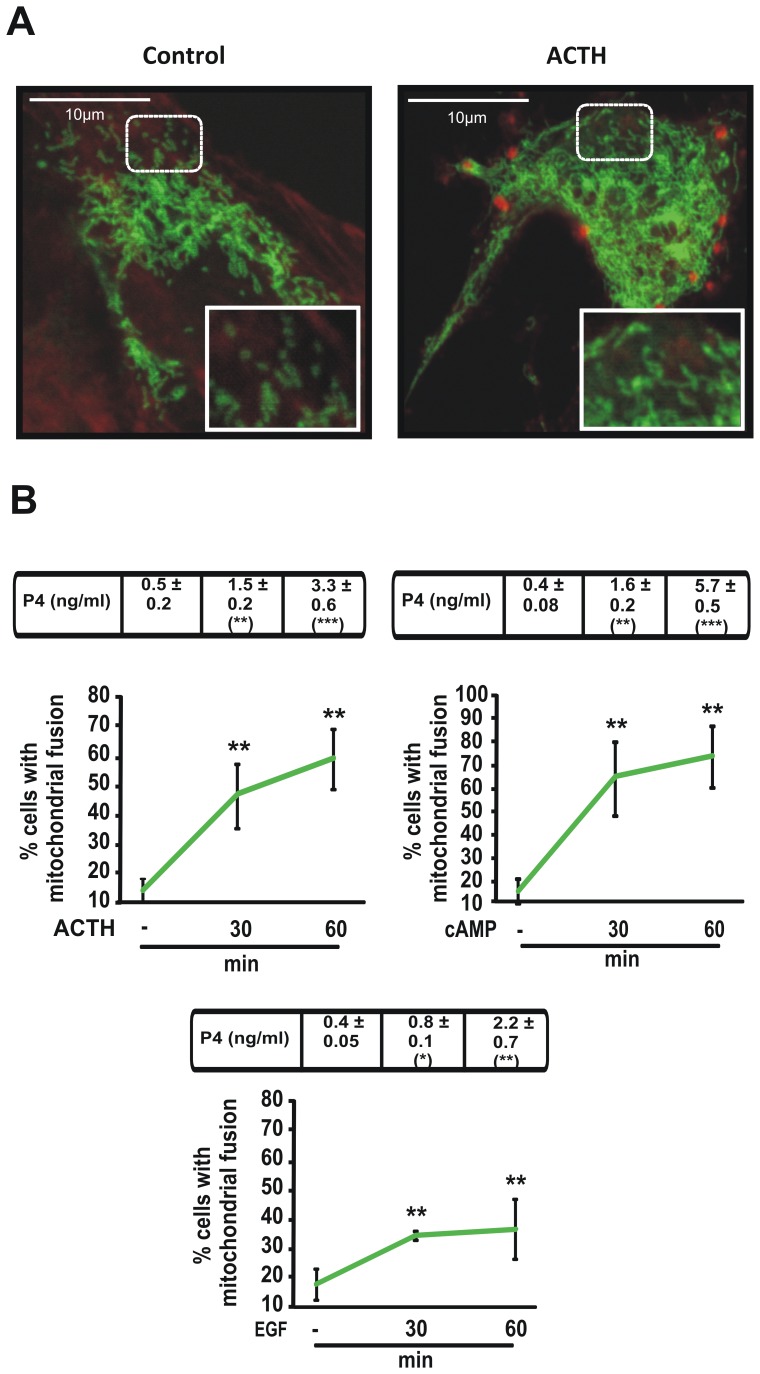
Hormonal stimulation causes an increase in fused mitochondria morphology in Y1 cells. A. Representative confocal images of Y1 cell line transfected with mtYFP. After 24 h transfection, cells were stimulated with or without ACTH (2 UI/ml) for 1 h. Cell morphology was visualized by actin red staining with the fluorescence dye Phalloidin–TRITC (1∶2000), incubated for 1 h at room temperature. Scale bar, 10 µm. **B.** After 24 h transfection with mt-YFP, Y1 cells were treated with or without ACTH (2 UI/ml), 8Br-cAMP (1 mM) or EGF (10 ng/ml), at the indicated times. See comments in Figure 1. Results of P4 measurement are indicated at the top of each graph as the means ± SEM of three independent experiments. *P<0.05 vs. control. ***P<0.001 vs. control. **P<0.01 vs. control.

We next quantified mitochondrial fusion kinetics under different stimuli such as hCG, cAMP or epidermal growth factor (EGF) in MA-10 Leydig cells (Figure 1C) and in Y1 adrenocortical cells (Figure 2 panels A and B). Remarkably, we observed a direct correlation between fused mitochondria and progesterone (P4) production in all cases. Moreover, when we used a slower steroidogenic stimulus, EGF, a delay in mitochondrial fusion was observed (Figure 1C lower panel).

### Mitochondrial Membrane Potential is Required for Steroid Synthesis and Mitochondrial Fusion

It has been previously shown that mitochondrial membrane potential (ΔΨm) affects steroid synthesis by blocking protein processing into mitochondria [27], [28]. Furthermore, dissipation of ΔΨm either by a protonophore (CCCP) or by a potassium-specific ionophore (valinomycin) abolishes mitochondrial fusion in the Hela cell line [29]; both drugs mediate the influx of cations until dissipation of ΔΨm and uncouple cellular respiration from ATP synthesis.

**Figure 3:**
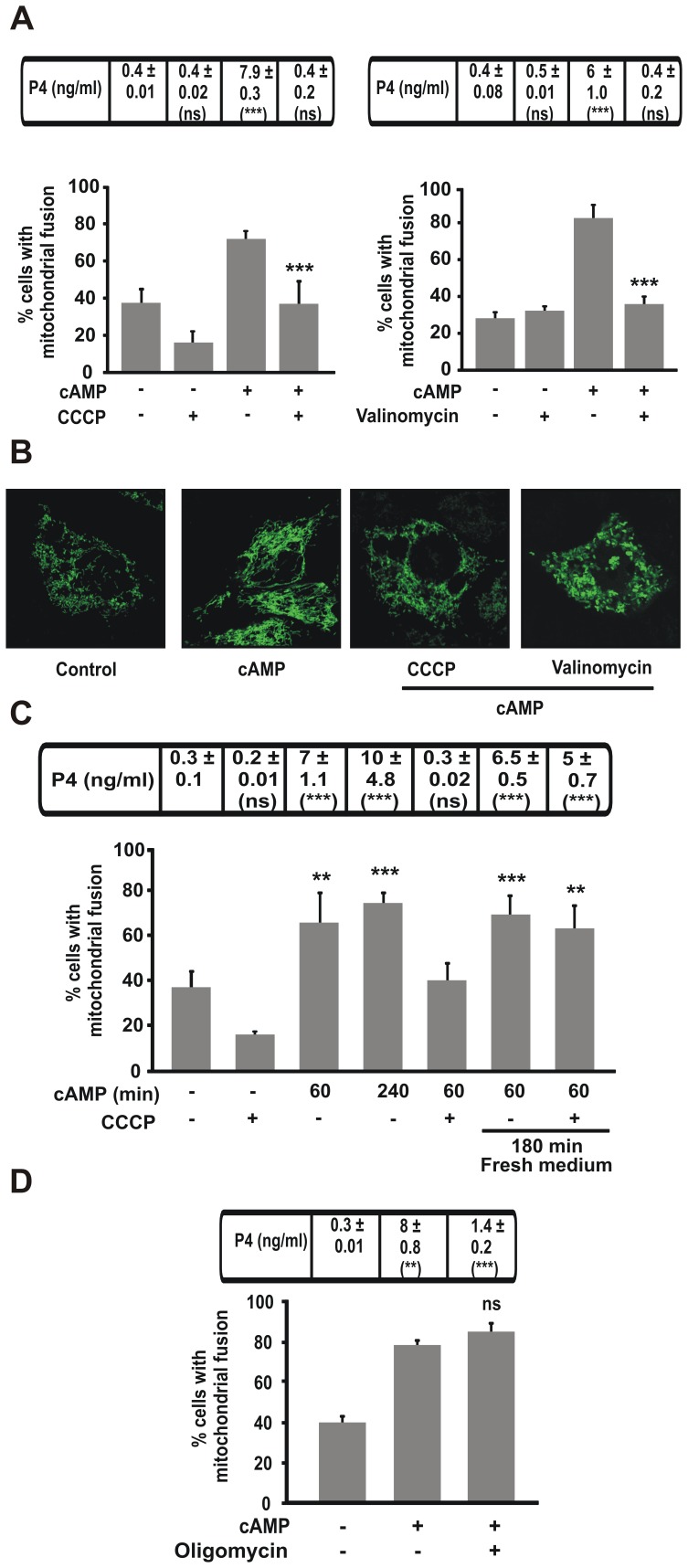
Mitochondrial membrane potential affects linearly fused mitochondria in MA-10 cells after hormonal stimulation. MA-10 cells were transfected with mt-YFP and 24 h post-transfection treated as described in each panel. Cells were scored and mitochondrial shapes were determinated. Quantitative analysis of fused mitochondrial shape is shown. **A.** Cells were treated with or without CCCP (5 µM) (left panel) or valinomycin (1 µM) (right panel) and 8Br-cAMP (1 mM) for 1 h. The results are expressed as the means ± SEM of three independent experiments. ***P<0.001 vs. control. **B.** Representative confocal images of mitochondrial shape in MA-10 cells treated with or without CCCP (5 µM) or valinomycin (1 µM) and 8Br-cAMP (1 mM) for 1 h. Scale bar, 10 µm. **C.** Cells were treated with or without CCCP (5 µM) and 8Br-cAMP (1 mM) for 1 h, or incubation with CCCP and 8Br-cAMP for 1 h followed by washout and 3 h recovery. The results are expressed as the means ± SEM of three independent experiments. **P<0.01 vs. control. ***P<0.001 vs. control. **D.** Cells were treated with or without oligomycin (1 µM) and 8Br-cAMP (1 mM) for 1 h. The results are expressed as the means ± SEM of three independent experiments. ns *P*>0.05 vs. 8Br-cAMP. Cellular medium was used to determine P4 production by RIA. Results of P4 measurement are indicated at the top of each graph as the means ± SEM of three independent experiments. ns *P*>0.05 vs. control. ***P<0.001 vs. control. **P<0.01 vs. control.

We therefore decided to evaluate the effect of different disrupters of mitochondrial membrane potential (CCCP and valinomycin) on the mitochondrial fusion and the steroid synthesis in steroidogenic cells under hormonal stimulation. Both compounds reduced mitochondrial fusion in cAMP-stimulated cells but not in control cells (Figure 3A). While CCCP-treated cells maintained a punctuated mitochondrial shape similar to that of control cells even after cAMP treatment (Figure 3B), valinomycin-treatment led to a different mitochondrial rearrangement, previously described as “swelling shape” (Figure 3B). This type of mitochondrial rearrangement has been previously associated with severe mitochondrial dysfunction [30]. In every case, CCCP inhibited mitochondria fusion thus suggesting that mitochondrial integrity is strictly necessary for mitochondrial fusion after cAMP treatment.

Finally, we removed CCCP and tested mitochondrial fusion and P4 as described in Figure 1. In this case, the inhibitory effect of CCCP on P4 production and mitochondrial fusion was equally reversed after the washout of this agent for 3 h. (Figure 3C). We also tested the mitochondrial ATP synthesis inhibitor, oligomycin and we observed no inhibitory effect on mitochondrial fusion (Figure 3D). These results suggest that mitochondrial fusion requires mitochondrial membrane potential but it is independent of ATP synthesis.

### Mitochondrial Fusion Promotes the Association between the Mitochondria and the Mitochondria-Associated Membrane (MAM) during Steroid Synthesis

**Figure 4:**
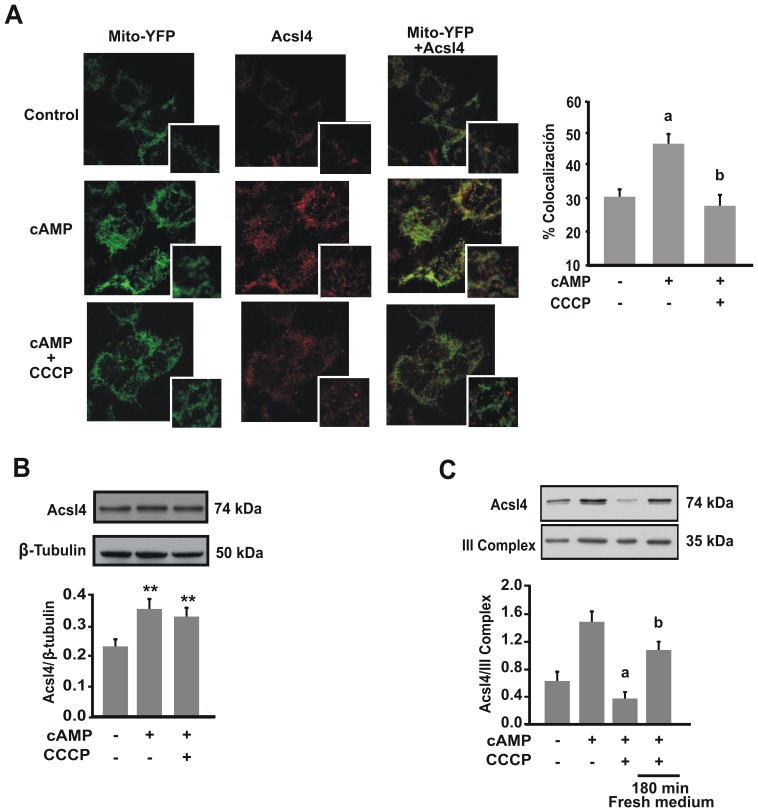
Hormonal stimulation allows an association between mitochondria and the MAM. A. After 24 hour transfection with mt-YFP (green), MA-10 cells were treated with or without CCCP (5 µM) and 8Br-cAMP (1 mM) for 1 h. Representative immunofluorescence to confocal section of co-localization between mitochondria and the MAM (Left panel). The MAM was stained with Acsl4 polyclonal antibody (red) followed by secondary antibody conjugated with Cy3 fluorofore as described in materials and methods. Signal overlap was quantified using MBF-Image J (Right panel). Pearsońs colocalization coefficients were calculated from three independent experiments and then converted to percentages. Data represent means ± SEM. a *P*<0.001 vs. control and b *P*<0.001 vs. 8Br-cAMP. **B.** MA-10 cells were treated with or without CCCP (5 µM) and 8Br-cAMP (1 mM) for 1 h. Total proteins were obtained and western blotting was performed. An image of a representative western blot is shown. Membranes were sequentially blotted with anti-Acsl4 and anti-β-tubulin antibodies. For each band, the optical density (OD) of the expression levels of Acsl4 protein was quantified and normalized to the corresponding β-tubulin protein. The relative levels of Acsl4 protein are shown. Data are presented as an average SEM of three independent experiments. **P<0.01 vs. control. **C.** MA-10 Cells were treated with or without CCCP (5 µM) and 8Br-cAMP (1 mM) for 1 h, or incubation with CCCP and 8Br-cAMP for 1 h followed by washout and 3 h recovery. Mitochondria were isolated and western blotting was performed. An image of a representative western blot is shown. Membranes were sequentially blotted with anti-Acsl4 and anti-OxPhos complex III core 2 subunit (III Complex) antibodies. For each band, the OD of the expression levels of Acsl4 protein was quantified and normalized to the corresponding III Complex protein. The relative levels of Acsl4 protein are shown. Data are presented as an average SEM of three independent experiments. a *P*<0.001 vs. 8Br-cAMP and b *P*<0.001 vs. control.

Acsl4 is an essential enzyme during steroid synthesis, which is located in a specialized compartment of the ER known as the mitochondria associated membrane (MAM) [31]. Since steroid synthesis requires both mitochondrion and Acsl4, we thought that an increased interaction between both components might enhance steroidogenesis. To determine the effect of cAMP stimulation on Acsl4 distribution, we evaluated the co-localization of this MAM enzyme with the mitochondrial one (mt-YFP). We observed that cAMP increased the co-localization between Acsl4 and mt-YFP (Figure 4A). Interestingly, inhibition of mitochondrial fusion with CCCP was sufficient to diminish the co-localization after the cAMP stimulus (Figure 4A). Since hormone stimulation increases Acsl4 levels [32], we followed the effect of CCCP on Acsl4 levels in total extracts. We observed no effect of CCCP on total protein levels, indicating that the increased co-localization with mt-YFP is not a result of an increase in Acsl4 levels (Figure 4B). In support of imaging experiments, we observed that Acsl4 levels are increased in isolated mitochondria under cAMP stimulation, an effect that was not observed under CCCP treatment (Figure 4C). Therefore, increased MAM-Acsl4 interaction with mitochondria correlated with mitochondrial fusion and steroid synthesis.

Together, these data strongly suggest that mitochondrial fusion is necessary to increase the association of mitochondria with MAM, a probably central event for an efficient transport of steroidogenesis intermediates.

### PKA and PTPs are Involved in Mitochondrial Fusion

**Figure 5:**
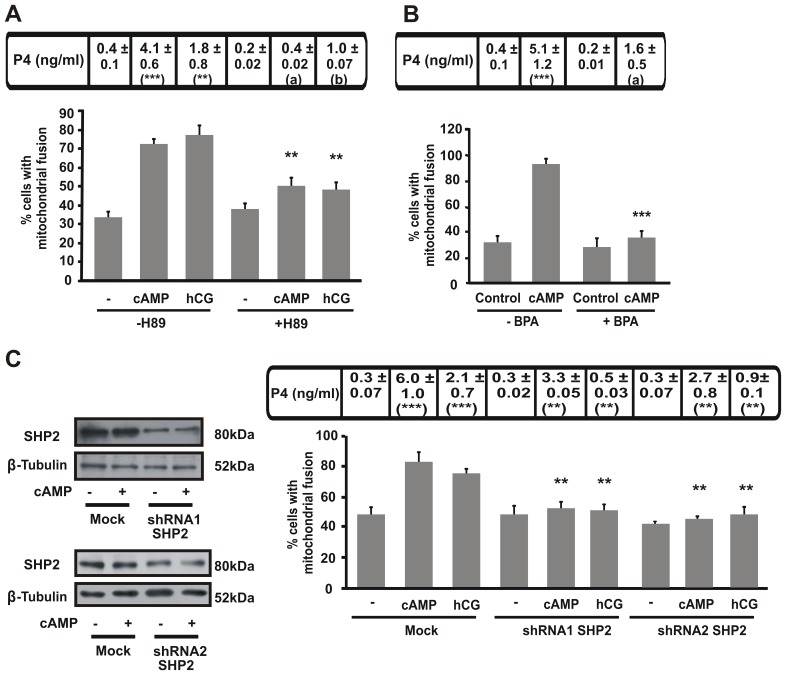
PKA activity induces mitochondrial fusion in steroid synthesis. MA-10 cells were transfected with mt-YFP and 24 h post-transfection treated as described for each panel. For each point, 100 cells were scored and mitochondrial shapes were classified. Quantitative analysis of fused mitochondria was shown. **A.** Cells were treated with or without H89 (20 µM) and stimulated with hCG (20 ng/ml) or 8Br-cAMP (1 mM) for 1 h. The results are expressed as the means ± SEM of three independent experiments. **P<0.01 vs. 8Br-cAMP without H89. **B.** Cells were treated with or without BPA (20 µM) and 8Br-cAMP (1 mM) for 1 h. The results are expressed as the means ± SEM of three independent experiments. ***P<0.001 vs. cAMP without BPA. **C.** Left panel: MA-10 cells were transfected with a plasmid containing different shRNA SHP2 (shRNA1 or shRNA2). After 48 h, total proteins were obtained and western blotting was performed. Membranes were sequentially blotted with anti-SHP2 and anti-β-tubulin antibodies. An image of a representative western blot is shown to assess shRNA1 and 2 knockdown efficiency. Right panel: cells were transfected with the plasmid combination, the mt-YFP-empty vector, mt-YFP-shRNA1 or mt-YFP-shRNA2 and after 48 h, cells were fixed and mitochondrial shape scored. The results are expressed as the means ± SEM of three independent experiments. **P<0.01 vs. cAMP mock. *P<0.05 vs. hCG mock. Cellular medium was used to determine P4. Results of P4 measurement are indicated at the top of each graph as the means ± SEM of three independent experiments. ***P<0.001 vs. control. **P<0.01 vs. control, a *P*<0.001 vs. cAMP without H89 or BPA and b *P*<0.01 vs. hCG without H89.

To evaluate the implication of PKA in mitochondrial fusion, we abolished PKA activity using H89, a specific inhibitor of this kinase. Cells treated with H89 significantly reduced the mitochondrial fusion observed after hormone or cAMP stimuli. As expected, steroid synthesis under both stimuli was diminished as well by H89 (Figure 5A). We then evaluated the effect of PTPs in mitochondrial fusion and steroid production. To perform this experiment, we used benzyl phosphonic acid (BPA), a PTPs inhibitor. We observed that BPA treatment reduced mitochondrial fusion and steroidogenesis in MA-10 cells under cAMP stimulation (Figure 5B).

We have recently identified SHP2 as a PTP necessary for steroid synthesis [10]. To further determine whether SHP2 is the PTP involved in the changes observed in mitochondrial morphology, we performed knockdown experiments using two specific short hairpin RNA (shRNA) directed against SHP2 protein. We found that knockdown of SHP2, by means of both shRNA, decreased steroid synthesis and mitochondrial fusion both under cAMP and hCG stimuli (Figure 5C), thus suggesting that mitochondrial fusion depends, at least in part, on SHP2 activity.

### Mitochondrial Fusion is Associated with the Efficient Localization of ERK1/2 into Mitochondria

**Figure 6:**
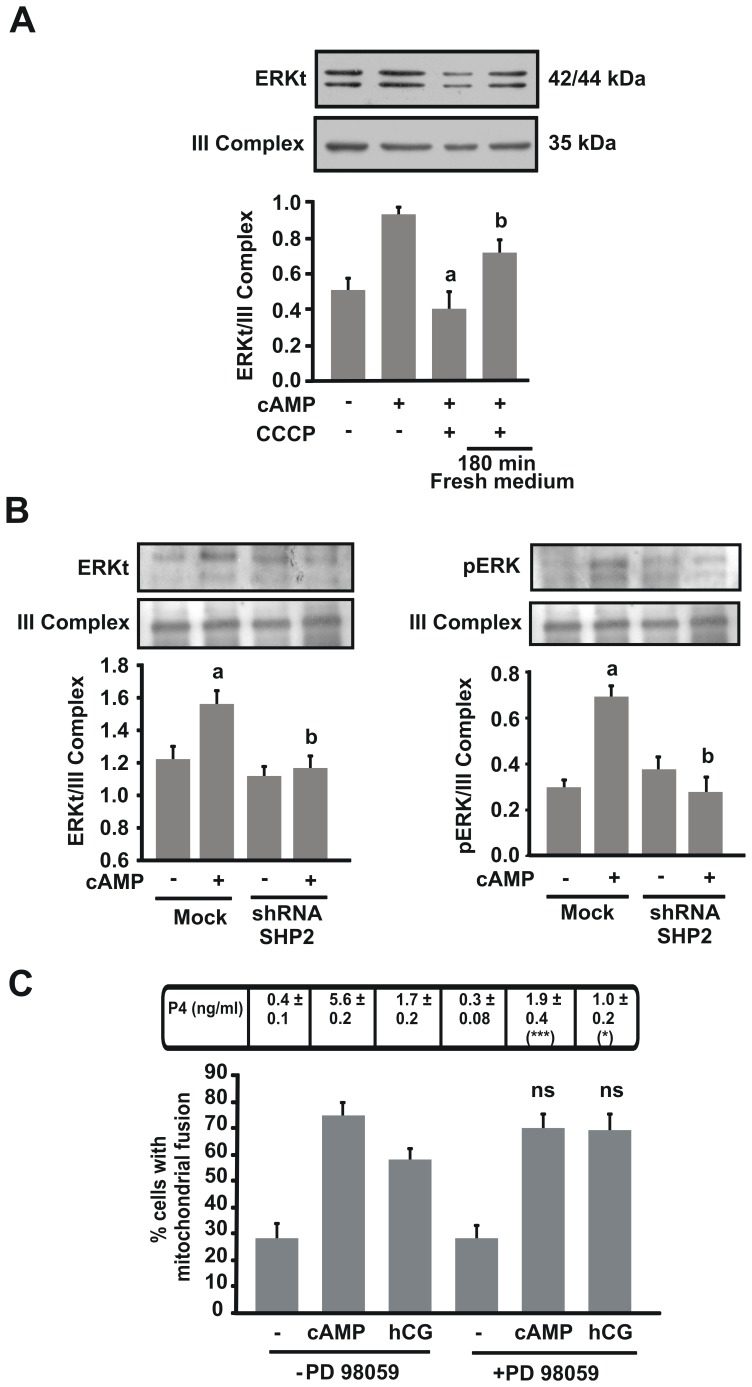
Mitochondrial fusion could allow access of ERK into mitochondria. A. MA-10 cells were treated with or without CCCP (5 µM) and 8Br-cAMP (1 mM) for 1 h, or incubation with CCCP and 8Br-cAMP for 1 h followed by washout and 3 h recovery. Mitochondria were isolated and western blotting was performed. An image of a representative western blot is shown (Top panel). Membranes were sequentially blotted with anti-ERKt and anti-III Complex antibodies. For each band, the OD of the expression levels of ERKt protein was quantified and normalized to the corresponding III Complex protein. The relative levels of ERKt protein are shown. Data are presented as an average SEM of three independent experiments. a *P*<0.001 vs. cAMP and b *P*<0.01 vs. control. **B.** Left panel: MA-10 cells were transfected with the shRNA1 SHP2 plasmid. After 48 h, mitochondria were isolated and western blotting was performed. An image of a representative western blot is shown. Membranes were sequentially blotted with anti-ERKt (total ERK) and anti-III Complex antibodies. For each band, the OD of the expression levels of ERKt protein was quantified and normalized to the corresponding III Complex protein. The relative levels of ERKt protein are shown. Right panel: an image of a representative western blot is shown. Membranes were incubated with a stripping buffer and blotted with anti-pERK (phosphorylated ERK). For each band, the OD of the expression levels of pERK protein was quantified and normalized to the corresponding III Complex protein. The relative levels of pERK protein are shown. The results are expressed as the means ± SEM of three independent experiments. a *P*<0.01 vs. Control and b *P*<0.01 vs. 8Br-cAMP mock. **C.** After 24 h transfection with mt-YFP, MA-10 cells were treated with or without PD98059 (50 µM) and stimulated with hCG (20 ng/ml) or 8Br-cAMP (1 mM) for 1 h. Cells were scored and mitochondrial shapes classified. The results are expressed as the means ± SEM of three independent experiments. ns *P*>0.05 vs. cAMP and hCG without inhibitor. Cellular medium was used to determine P4 production by RIA. Results of P4 measurement are indicated as the means ± SEM of three independent experiments. ***P<0.001 vs. cAMP without PD. *P<0.05 vs. hCG without PD.

ERK1/2 activity is critical to achieve optimal steroidogenesis [5] and, under hormonal stimulation, it is found in the mitochondria [5]. To determine whether mitochondrial fusion is associated with ERK1/2 localization in mitochondria, we performed experiments using CCCP and a shRNA for SHP2. First, cells were treated with CCCP under cAMP stimulation. Location of ERK in mitochondria was observed under cAMP stimulus and this effect was diminished by CCCP. Interestingly, in CCCP-recovered cells, ERK1/2 expression returned to the levels observed in non-CCCP-treated cells (Figure 6A). In addition, after SHP2 knockdown, recruitment of ERK1/2 to mitochondria and its phosphorylation status were significantly decreased (Figure 6B). These experiments indicate that SHP2 activity not only affects mitochondrial fusion to recruit ERK1/2 in mitochondria but could also regulate its phosphorylation in this organelle.

We next analyzed the role of ERK1/2 phosphorylation in mitochondrial fusion. We inhibited ERK1/2 phosphorylation, using PD98059, a MEK inhibitor. Treatment with the inhibitor had no effect on fused mitochondria under hCG and cAMP stimulation (Figure 6C). This result indicates that mitochondrial fusion is an event upstream ERK1/2 phosphorylation.

### Steroidogenesis Requires Mitofusin 2

**Figure 7:**
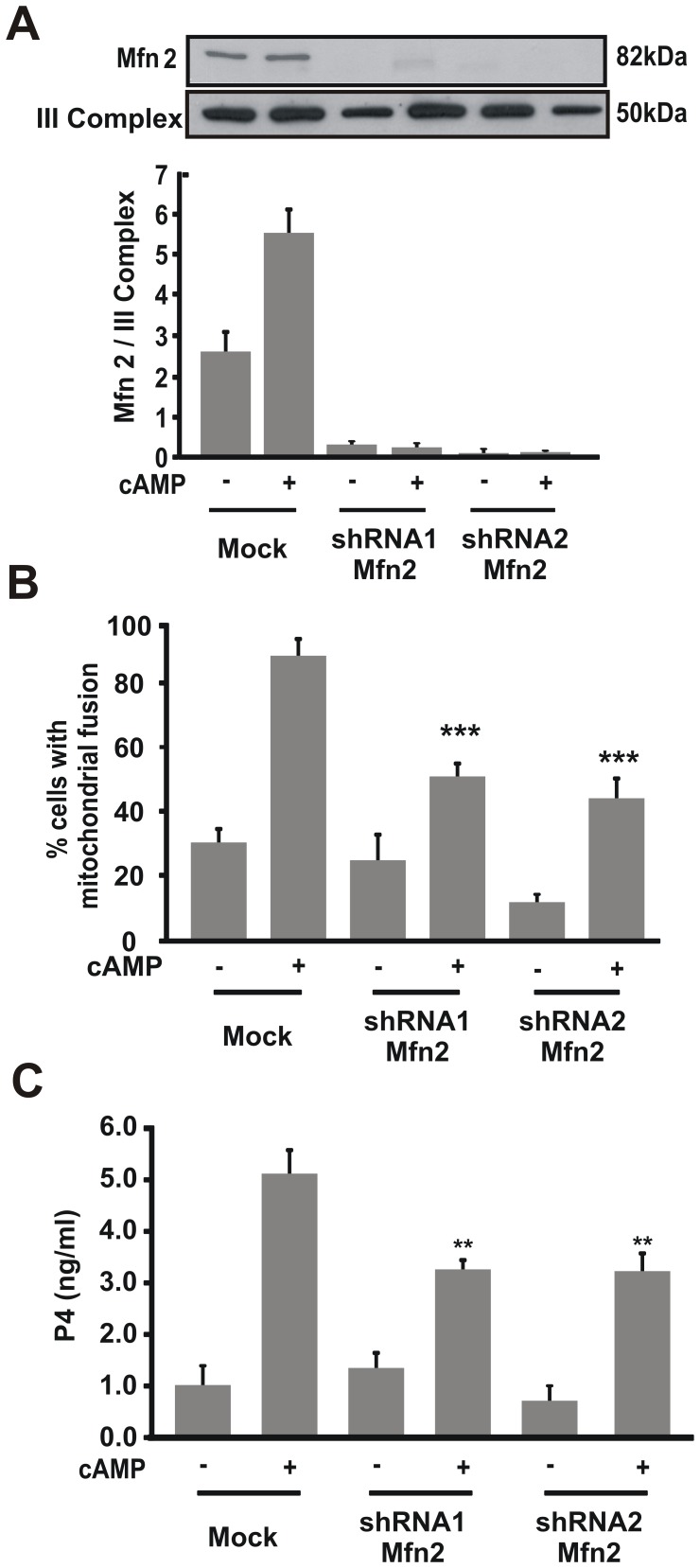
Mfn2 protein is necessary for steroid synthesis. MA-10 cells were transfected with a plasmid containing different shRNA Mfn2 (shRNA1 or shRNA2). After 48 h, cells were stimulated with 8Br-cAMP (0.5 mM) for 1 h. **A.** Isolated mitochondrial proteins were obtained and western blotting was performed. Membranes were sequentially blotted with anti-Mfn2 and anti-III Complex antibodies. An image of a representative western blot is shown. For each band, the OD of the expression levels of Mfn2 protein was quantified and normalized to the corresponding III Complex protein. The relative levels of Mfn2 protein are shown. **B.** Cells were fixed and scored as previously described. Quantitative analysis of mitochondrial fusion is shown. The results are expressed as the means ± SEM of three independent experiments. **P<0.01 vs. cAMP mock. **C.** P4 levels were determined by RIA in the incubation media. Data represent the means ± SEM of three independent experiments and expressed as ng/ml. **P<0.01 vs. 8Br-cAMP mock.

Mfn2 protein has been proposed as an important mitochondrial protein involved in the fusion formation of the mitochondrial network [33], [34]. To study the role of Mfn2 in steroid synthesis, we generated two specific shRNA directed against Mfn2. First, we verified the correct knockdown of Mfn2 using both shRNA constructs. Both shRNA-Mfn2 significantly decreased the levels of this protein (Figure 7A). Next, we evaluated the effect of Mfn2 knockdown on mitochondrial fusion and steroid synthesis. We observed that knockdown of Mfn2 reduced mitochondrial fusion and significantly decreased steroid production under cAMP stimulation (Figure 7B and C). Thus, we concluded that Mfn2 participates in steroid synthesis through mitochondrial fusion.

### Mitofusin 2 Expression is Regulated by cAMP and hCG in MA-10 Cells

**Figure 8:**
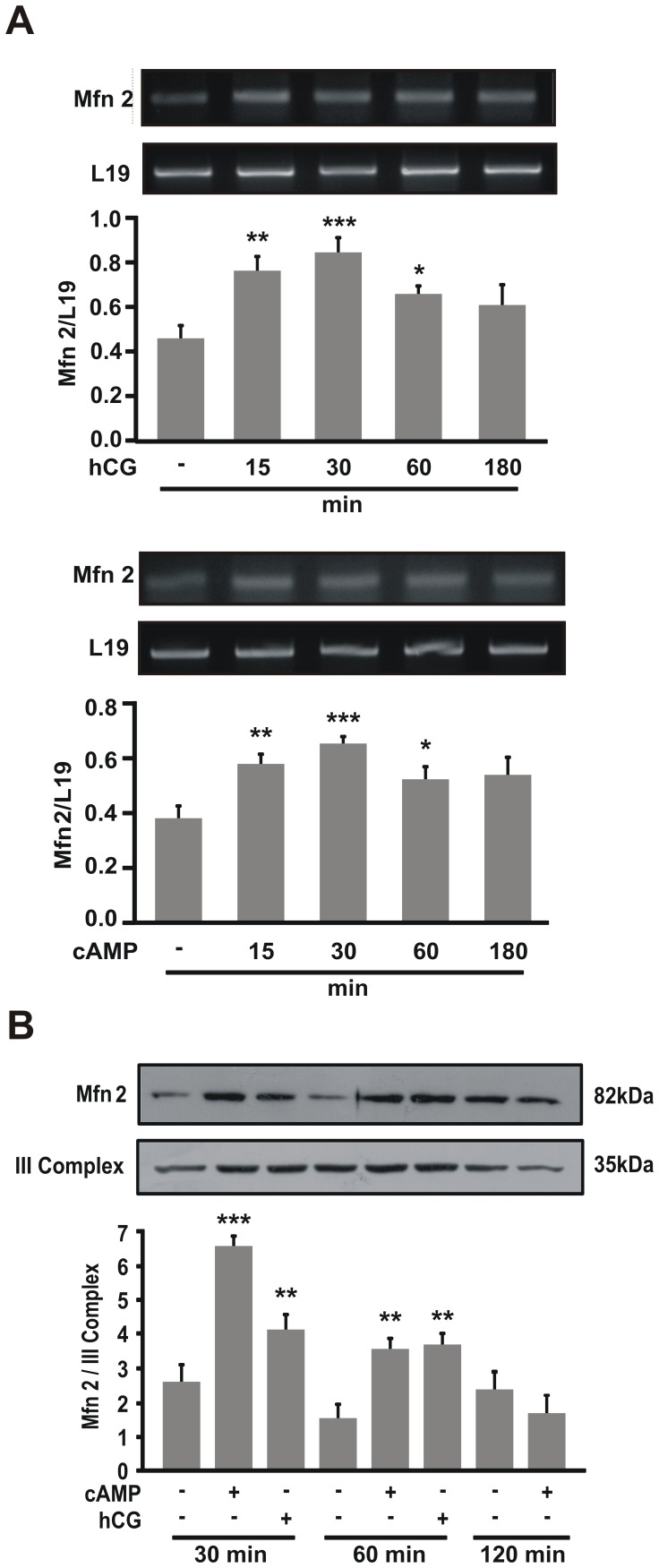
Hormonal stimulation induces Mfn2 expression. MA-10 cells were treated with or without hCG (20 ng/ml) or 8Br-cAMP (1 mM) for the indicated times. **A.** Total RNA was isolated; reverse-transcribed, and subjected to semi-quantitative PCR using specific primers for Mfn2 and L19 cDNA as loading controls. PCR products were resolved in ethidium bromide-stained agarose gels. The figure shows representative gels. For each band, the OD of the expression levels of Mfn2 was quantified and normalized to the corresponding L19 abundance. The relative levels of Mfn2 are shown. The results are expressed as the means ± SEM of three independent experiments. **P<0.01 vs. control, ***P<0.001 vs. control, *P<0.05 vs. control. **B.** Mitochondria were isolated and western blotting was performed. An image of a representative western blot is shown. Membranes were sequentially blotted with anti-Mfn2 and III Complex antibodies. For each band, the OD of the expression levels of Mfn2 protein was quantified and normalized to the corresponding III Complex protein. The relative levels of Mfn2 protein are shown. The results are expressed as the means ± SEM of three independent experiments. ***P<0.001 vs. control. **P<0.01 vs. control.

Although several lines of evidence support different regulations of Mfn2 [14], hormonal regulation of Mfn2 levels in cellular differentiation has not been demonstrated. Thus, we analyzed the effects of hCG and cAMP on Mfn2 expression. We observed that both treatments induced Mfn2 mRNA levels (Figure 8A). Remarkably there is a rapid mRNA induction at 30 min with cAMP and hCG. A longer time of stimulation did not show significant differences compared to control cells. We further analyzed protein expression levels under hCG and cAMP stimulation. The same stimulation pattern was observed in protein expression (Figure 8B). Together with the experiments described in Figure 7, these results demonstrate for the first time that Mfn2 expression is hormonally regulated and that mitochondrial fusion is stimulated and required for steroidogenesis.

## Discussion

Herein, we showed that mitochondrial fusion is able to regulate specialized cellular functions as steroid synthesis. We found that mitochondrial fusion precedes and is required for steroid biosynthesis. In this work we propose a novel mechanism in which mitochondrial fusion is determined by a cross-talk between serine/threonine phosphorylation and tyrosine dephosphorylation events triggered by hormonal stimulation. This mechanism involves PKA and SHP2 activity in the mitochondrial fusion changes. Furthermore, we demonstrate that mitochondrial fusion allows the localization of key proteins for steroid synthesis such as ERK1/2 and Acsl4 in mitochondria to achieve steroidogenesis.

We also showed for the first time that Mfn2 expression is hormonally regulated and is needed for steroidogenesis. Together, we provide compelling evidence demonstrating not only the requirement but also the molecular bases that link mitochondrial fusion with the proper activation of full steroidogenesis.

### The Events that Trigger Changes in Mitochondria Fusion during Steroidogenesis

In the present study, we report that mitochondrial fusion and steroidogenesis depend on the mitochondrial membrane potential under cAMP stimulation. The data presented here show that intracellular ATP levels are not a requirement for mitochondrial fusion, since oligomycin inhibits steroid production but does not affect mitochondrial fusion in stimulated cells. In this regard, it has been proved that GTP and not ATP is required for *in vitro* mitochondrial fusion in yeast [35]. It is known that arachidonic acid (AA) is required for gene expression of StAR (Steroidogenic Acute Regulatory) protein, key in steroidogenesis [36]. We have previously demonstrated that there is an export of AA from the mitochondria that is dependent of ATP synthesis [37]. Therefore, oligomycin inhibits steroid biosynthesis due to the decrease in AA export. Since oligomycin had no effect on mitochondrial fusion, it strongly suggests that the mechanism of AA export and StAR synthesis is downstream of mitochondrial fusion. Given that ATP is required for steroid synthesis [37], our results suggest that mitochondrial fusion is a previous step in steroidogenesis.

It is known that phospho/dephosphorylation events are obligatory in cholesterol transport to the inner mitochondrial membrane, and that PKA is one of the proteins required for this process [4]. We determined that mitochondrial fusion depends on PKA activity. Furthermore, it has been recently proposed that PKA increases mitochondrial fusion events and cell survival [24]. Also, PTPs activity is required for steroid synthesis [38]. We have recently identified SHP2 as a PTP that participates in the cAMP-dependent activation of steroidogenesis [10]. Its activity is modulated by PKA phosphorylation after ACTH challenge in adrenocortical cells [39]. Our data show that PTPs are involved in the mitochondrial switch to the fusion shape and establish that SHP2 is the PTP that participates in the mitochondrial fusion mechanism.

Steroidogenic hormones promote a notable change in cell shape, implicating reorganization of the actin cytoskeleton, preceding steroid biosynthesis and secretion [40], [41]. Paxillin is a focal adhesion protein that is rapid tyrosine dephosphorylated in the Y1 adrenocortical cells by ACTH/cAMP [42]. Moreover, paxillin is dephosphorylated by SHP2 in MCF-7 cells to regulate cell motility [43]. Thus, it is conceivable to consider that SHP2 activity, which in turn dephosphorylates paxillin, modulates the actin cytoskeleton with a direct relationship with mitochondrial subcellular distribution and fusion.

### The Localization of Proteins to the Mitochondria Depends on Changes in Mitochondria Dynamics

Reorganization of organelles and contact between membranes can be a primary process in steroid production and secretion through the plasma membrane. The localization of several enzymes is the clue to ensure appropriate steroidogenesis rates. In steroidogenic cells, Acsl4 is induced after hormonal stimulation [32] and is localized in the ER [30], particularly in the MAM [31]. Interestingly, we detected that Acsl4 is not associated with mitochondria if mitochondrial fusion is inhibited. Restored mitochondrial fusion re-localized this protein in the mitochondrial context. On the other hand, it has been proposed that Acsl4 could have a role in MAM enhancing the mitochondrial and ER membrane fluidity [44]. These data suggest that the increased contacts between Acsl4 and mitochondria during mitochondrial fusion could allow steroid movement between these two organelles. In addition, we demonstrated that ERK1/2 locates to the mitochondria after hCG/8Br-cAMP. Furthermore, ERK1/2 mobilization is dependent on SHP2-mediated mitochondrial fusion.

### The Molecular Signal that Triggers Mitochondrial Fusion during Steroidogenesis

Mitochondrial fusion is carried out by mitofusins, among which Mfn2 is mainly involved [33]. We added new evidence on the correlation between mitochondrial fusion and steroid production. Our results indicate that fusion *per se* is obligatory for steroid biosynthesis and rule out any other unspecific effect of mitochondrial fusion inhibition on steroidogenesis.

We show for the first time that Mfn2 mRNA and protein levels are modulated by hCG and cAMP in this cellular type. This suggests that mitochondrial fusion could forward an increase in cellular energy when abrupt changes, such as steroid hormone synthesis, are required.

In summary, here we provide a large body of evidence about how mitochondrial fusion operates in steroidogenic cells. More significantly, our results provide a framework to understand how liposoluble steroid hormones can shift between mitochondria and the MAM, by a non-vesicular traffic, to reach at plasma membrane without moving through the cytoplasmic hydrophilic milieu.

## Materials and Methods

### Materials

Waymouth MB752/1 cell culture media, acrylamide, bis.acrylamide, agarose, mEGF, BSA, oligomycin and carbonyl cyanide m-chlorophenyl hydrazone (CCCP) were purchased from Sigma Chemical Co. (St. Louis, MO, USA). Ham-F10 cell culture medium, sera, antibiotics, trypsin-EDTA, TriZol reagent, and Lipofectamine 2000 were from Life Technologies, Inc. (Gaithersburg, MD, USA). Polyclonal antibodies against Acsl4 were previously obtained in our laboratory [32], [45]. Anti-tubulin monoclonal antibody was purchased from Upstate (Lake Placid, NY, USA). Electrophoresis supplies, polyvinylidendifluoride membrane (PVDF), and secondary antibody (horseradish peroxidase conjugated goat antibody) were from Bio-Rad Laboratories Inc. (Hercules, CA, USA). M-MLV reverse transcriptase (RT), GoTaq DNA polymerase, RNAsin inhibitor, RNase-free DNase RQ, and other molecular biology reagents were purchased from Promega (Madison, WI). Oligonucleotides were obtained from Invitrogen (Carlsbad, CA, USA). Bisacetoxymethyl ester (AM), derivative of benzylphosphonic acid (BPA) benzylphosphonic acid-(AM)2, [(BPA-(AM)2)] was purchased from BIOMOL International. Oligomycin and carbonyl cyanide m-chlorophenyl hydrazone (CCCP) were from SIGMA Chemical Co. (St. Louis, MO, USA). Sterile and plastic material for tissue culture was from Orange Scientific. All other reagents were of the highest grade available.

### Cell Culture

The MA-10 cell line is a clone strain of mouse Leydig tumor cells that produces progesterone rather than testosterone as the main steroid [46]. MA-10 cells were generously provided by Mario Ascoli from the University of Iowa, College of Medicine (Iowa City, IA) and were handled as described previously [5]. The growth medium consisted of Waymouth MB752/1 containing 1.1 g/l NaHCO3, 20 mM HEPES, 50 g/ml gentamicin, and 15% heat-inactivated horse serum.

Murine Y1 cell line is a clone strain of adrenocortical tumor cells, generously provided by Bernard Shimmer (University of Toronto, Toronto, Canada) [47], were maintained in Ham-F10 medium, supplemented with 12.5% heat-inactivated horse serum and 2.5% heat-inactivated fetal bovine serum, 1.2 g/liter NaHCO3, 200 IU/ml penicillin, and 200 g/ml streptomycin sulfate and were handled as described previously [32]. Flasks and multi-well plates were maintained at 36 C in a humidified atmosphere containing 5% CO2.

Human chorionic gonadotropin (purified hCG, batch CR-125 of biological potency 11900 IU/mg; gift from NIDDK, NIH) was used to treat the cells (10 or 20 ng/ml) for the times indicated. 8Br-cAMP (Sigma-Aldrich, St. Louis, MO), a permeable analog of cAMP, was used to treat the cells (0.5 or 1 mM) for the times indicated. mEGF stimulation was performed in culture medium containing 0.1% BSA to treat the cells (10 ng/ml) for the times indicated.

### shRNA Vectors

To obtain the knockdown plasmid, we used two different 19-bp DNA fragment of the mouse SHP2 named shRNA1-GATTCAGAACACTGGGGAC and shRNA2-GAGTAACCCTGGAGACTTC in the adequate frame shift to generate a shRNA, which the cells process to generate a functional and active small interfering RNA (siRNA) directed against the murine SHP2. A Blast search confirmed that the sequence specifically recognizes mouse SHP2. The insert was cloned in the pSUPER.retro.puro vector (Oligoengine) and the obtained SHP2 shRNA constructs verified by nucleotide sequencing (Macrogen) and used as previously reported [10].

For Mfn2 analysis, we used two different 19-bp DNA fragments of the mouse Mfn2 named shRNA1-ACACATGGCTGAAGTGAAT (nucleotides 366–380) and shRNA2-CTGGACAGCTGGATTGATA (nucleotides 934–948) obtained as described above.

### Plasmid Transfection

MA-10 cells were transiently transfected. One day before transfection, MA-10 cells (5×10^5^ cells/well) were grown up to 80% confluence onto cover glasses (12 mm) into 24-well plates. Transfection was performed with either 0.4 µg of plasmid containing the mitochondrial target sequence and the yellow fluorescence protein (mt-YFP, Clontech), 0.4 µg mt-YFP plus 0.4 µg of pSUPER.retro plasmid or 0.4 µg mt-YFP plus 0.4 µg of the plasmid with the corresponding shRNA in Opti-MEM medium and 2 µl Lipofectamine 2000 reagent (Invitrogen, Carlsbad, CA) according to the instructions of the manufacturer. Cells were placed into normal culture medium 6 h after transfection and grown for further 24 h. The cells were then used as described in the respective figures. Transfection efficiency was approximately 30% as estimated by counting fluorescent cells transfected with the pRc/CMVi plasmid containing the enhanced form of green fluorescent protein.

### Mitochondrial Morphology Assay in Cell Culture and Microscopy

MA-10 and Y1 cells cultured on poly-D-lysine-coated cover glasses (12 mm) were transfected with mt-YFP using Lipofectamine 2000 and analyzed 24 h later. Cells with the indicated mitochondrial morphology characterized as tubular fusion-shape mitochondria were quantified. More than a hundred cells were counted manually in at least four distinct optical fields.

Mitochondrial morphology was scored by reference image-based model. For the former, coded images were assigned two different shapes by comparison to a set of reference images of mitochondrial clustering/punctuated and elongation/fusion [26].

Cell morphology was visualized by actin red staining with the fluorescence dye Phalloidin–TRITC (1∶2000), incubated for 1 h at room temperature.

The images were visualized using an Olympus BX50 epifluorescence microscope coupled to a Cool/Snap Proof Color PM-c35 camera. Pictures were imported into Microsoft PowerPoint for presentation.

### Immunofluorescence Analysis

MA-10 or Y1 cells were grown to approximately 60% confluence on poly-D-lysine-coated cover glasses (12 mm). After treatments, cells were fixed with 4% paraformaldehyde (PFA) in PBS for 10 min at room temperature and permeabilized with 0.01% Triton X-100 for 10 min at 4°C. After several washes with 1% PBS-0.05% Tween-20, cells were blocked with 1% albumin in PBS-0.05% Tween-20 for 60 min at room temperature. Cells were incubated with the corresponding primary antibody overnight at 4°C [anti-Acsl4 polyclonal IgG (1∶5000)]. After several washes, cells were incubated for 1 h at room temperature with the corresponding secondary goat Cy3- conjugated antibody (1∶400) directed against rabbit immunoglobulins. Coverslips were mounted onto the slides using Fluorsave antifade reagent (Calbiochem, CA) followed by examination using a Zeiss LSM 510 laser-scanning confocal microscope.

### Electron Microscopy

MA-10 cells were incubated in the absence of presence of 8Br-cAMP 1 mM for 1 h. After treatment, cells were fixed in 3% glutaraldehide-phosphate buffered saline 0.1 M, pH 7.5 for 1 h. The cells were washed with phosphate buffered saline, detached from the culture dishes with a rubber policeman in the presence of 20% ethanol and collected for centrifugation. Cells pellets were maintained in fresh fixation buffer overnight at 4°C. Then, pellets were post-fixed in 1.5% of osmium tetroxide (Sigma-Aldrich) and embedded in Epoxi Embedding Medium Kit (Sigma-Aldrich). After that, were cut into thin sections, mounted on copper grids and stained with lead citrate. All sections were observed with an electronic microscope Zeiss 109T and microphotographies were taken with a ERlangshen ES 1000W Model 785-GATAN camera.

### Protein Quantification and Western Blot

Protein was determined by the method of Bradford [48] using BSA as a standard.

Total or mitochondrial proteins (30 µg) were separated on 10% or 12% SDS-PAGE and electro-transferred to PVDF membranes as described previously [49]. Membranes were then incubated with 5% fat-free powdered milk in 500 mM NaCl, 20 mM Tris-HCl (pH 7.5), and 0.5% Tween 20 for 60 min at room temperature with gentle shaking. The membranes were then rinsed twice in 500 mM NaCl, 20 mM Tris-HCl (pH 7.5), and 0.5% Tween-20 and incubated overnight with the appropriate dilutions of primary antibody rabbit polyclonal anti-Acsl4 (1∶5000), rabbit polyclonal anti-ERK1/2t (1∶1000), rabbit polyclonal anti-pERK1/2 (1∶5000), rabbit polyclonal anti-Mfn2 (1∶1000); mouse monoclonal anti-SHP2 (1∶5000), mouse monoclonal anti-β-tubulin (1∶5000) and anti-OxPhos complex III core 2 subunit (Invitrogen, Carlsbad, CA) antibody, named III-complex (1∶10000), and immunoreactive bands were detected using enhanced chemiluminescence (GE Healthcare, Buckinghamshire, UK).

### Isolation of Mitochondria

Mitochondria were isolated as described previously [37]. Briefly, MA-10 cell cultures were washed with PBS, scraped in 10 mMTris-HCl (pH 7.4), 250 mM sucrose, 0.1 mM EDTA, 10 µm leupeptin, 1 µm pepstatin A, and 1 mM EGTA (buffer A), homogenized with a Pellet pestle motor homogenizer (Kimble Kontes, Vineland, NJ), and centrifuged at 600×*g* for 15 min. The supernatant obtained was centrifuged at 10000×*g* for 15 min and rendered a mitochondrial pellet that was washed once with buffer A and resuspended in 10 mM Tris-HCl (pH 7.4), 10 µm leupeptin, 1 µm pepstatin A, and 1 mM EGTA. As described elsewhere [31], mitochondrial fraction isolated by this technique shows some content of proteins localized in the MAM.

### RNA Extraction and Semi-quantitative RT-PCR

Total RNA from the different treatment groups was extracted using Tri-Reagent TriZol reagent following the manufacturer’s instructions (Molecular Research Center, Inc. Cincinnati). The reverse transcription and PCR analyses were carried out using 2 µg of total RNA for cell lines or purified Leydig cells, respectively. The cDNAs generated were further amplified by PCR under optimized conditions using the primer pairs listed below. The primers used for isolation and amplification of the Mfn2 (amplicon size 120 bp) were: sense primer, 5′-GCACCGCCATATAGAGGAAGG-3′ and antisense primer, 5′-CGCACAGACACAGGAAGAAGG-3′. Primers specific for a 405-bp segment of ribosomal protein L19 were used as housekeeping genes [50]. For the comparison of the amount of amplified Mfn2 produced from different RNA samples, the amplified L19 product of each sample was used as an internal standard, using the sense primer, 5′-GAAATCGCCAATGCCAACTC-3′, and the antisense primer, 5′-TCTTAGACCTGCGAGCCTCA-3′ [51]. The reaction conditions were one cycle of 94°C for 5 min, followed by 30 cycles for Mfn2 or 23 for L19 of 94°C for 30 sec, 56°C for 30 sec, and 76°C for 90 sec. The number of cycles used was optimized for each gene to fall within the linear range of PCR amplification. PCR products were resolved on a 2% (wt/vol) agarose gel containing 0.5 µg/ml of ethidium bromide to determine the molecular sizes of the Mfn2 and L19 amplicons. The gel images were acquired with the GelPro analyzer (IPS, North Reading, MA). The levels of the Mfn2 and L19 mRNA were quantitated using a computer-assisted image analyzer (ImageQuant 5.2) and the PCR results for each sample were normalized by L19 mRNA as an internal control.

### Radioimmunoanalysis (RIA) and Statistics

Progesterone production in cell culture media was measured by RIA as described previously [45]. Data are expressed as ng/ml.

Statistical significance was determined using the Student’s *t* test or analysis of variance (ANOVA) followed by the Student-Newman-K.
